# Hand movements respond to any motion near the endpoint

**DOI:** 10.3758/s13414-022-02471-w

**Published:** 2022-03-25

**Authors:** Emily M. Crowe, Jeroen B. J. Smeets, Eli Brenner

**Affiliations:** Department of Human Movement Sciences, Institute of Brain and Behavior Amsterdam, Amsterdam Movement Sciences, Vrije Universiteit Amsterdam, 1081 BT Amsterdam, The Netherlands

**Keywords:** Online control, Interception, Perturbation, Surface interpretation

## Abstract

Hand movements are pulled in the direction of motion near their planned endpoints. Is this an automatic response to motion signals near those positions, or do we consider what is moving? To find out, we asked participants to hit a target that moved rightward across a patterned surface when it reached an interception zone that was indicated by a circle. The circle was initially at the center of a square. The square was either filled, occluding the patterned surface (tile), or open, such that the patterned surface was not occluded (frame). The square briefly moved leftward or rightward shortly after the target appeared. Thus, participants were either aiming to hit the target on the surface that moved (the tile) or to hit the target on the patterned surface that did not move. Moving the two types of squares produced very similar local motion signals, but for the tile this could be interpreted as motion of an extended surface, while for the frame it could not. Motion onset of the two types of squares yielded very similar responses. Increasing the size of the square, and thus the eccentricity of the local motion signal, reduced the magnitude of the response. Since this reduction was seen for both types of squares, the surface on which the interception zone was presented was clearly not considered. We conclude that the response is driven by local motion signals near the endpoint of the action without considering whether the local surface is moving.

## Introduction

We rely on the visual information in our surrounding to successfully interact with the world. The visual scene that is projected onto the retina is organized into coherent regions: the objects and surfaces that constitute our environment. Gestalt psychologists have identified many criteria for segmenting visual scenes into regions, such as similarity, proximity, closure, and connectedness (for reviews, see Peterson & Kimchi, 2013; Rock & Palmer, 1990; Wagemans et al., 2012). Each region of a visual scene could be interpreted as an object or as a surface behind an object (Rubin, 1921). Such interpretation is important for both object recognition and interacting with objects in our environment.

In daily life, we often make visually guided reaching movements to intercept or pick up items. Such movements need to be adjusted when something changes such as the item you are reaching for falling over or you yourself shifting because someone bumped into you. It is well documented that the moving arm quickly follows target displacements (reviewed by Smeets, Oostwoud Wijdenes, & Brenner, 2016). It is also known that the moving arm quickly follows motion onsets in the surrounding area (e.g., Brenner & Smeets, 1997; Gomi, Abekawa, & Nishida, 2006; Saijo, Murakami, Nishida, & Gomi, 2005; Whitney, Westwood, & Goodale, 2003; Zhang, Brenner, Duysens, Verschueren, & Smeets, 2018, Zhang, Brenner, Duysens, Verschueren, & Smeets, 2019), in particular if they occur near the anticipated movement endpoint (e.g., Brenner & Smeets, 2015; Crowe, Smeets, & Brenner, 2021). We propose that this following response is due to shifting the planned movement endpoint in the direction of the motion onset (Crowe et al., 2021). The position of the planned endpoint obviously only changes if the motion onset pertains to the surface at which the movement is supposed to end, so the interpretation of the scene as consisting of surfaces is relevant. We therefore wondered whether the following response is only driven by motion of the surface on which the movement is supposed to end, or whether it is driven directly by any motion signals near the movement endpoint.

To find out whether the response considers whether the local surface is moving, we compared conditions that produce similarly localized motion signals but have different surface interpretations. We compared the responses to two types of squares (frame and tile) of three sizes that were always initially centerd on the interception zone that was indicated by a circle. The frame did not cover a patterned surface, so the movement ended on the patterned surface. In contrast, the tile covered this patterned surface such that the movement ended on the tile. The squares shifted to the left or right at a given moment after the moving target appeared, so the motion signal generated by the shift was restricted to the squares’ (vertical) edges. For the larger squares, the motion signal is farther from the target, so if only local motion information is considered, the magnitude of the response should decrease with size (Crowe et al., 2021). If the response considers whether the local surface is moving, the predictions differ between the frame and tile. For a frame there is no reason to interpret the motion as being near the movement endpoint, especially if the frame is large. In contrast, for a tile the whole area near the movement endpoint belongs to the moving surface, irrespective of the size of the tile. Therefore, if the response considers whether the local surface is moving, one would expect a much smaller response for the frame than for the tile, especially when they are large.

## Method

### Participants

Twelve participants (11 right-handed; age 27.9 ± 2.3 years; graduate students; convenience sample based on the heuristics justification – Lakens, 2021) volunteered to take part in the experiment. The study was approved by the local ethics committee (Ethics Committee of the Vrije Universiteit Amsterdam) in accordance with the tenets of the Declaration of Helsinki.

### Set-up

The experiment was conducted in a normally illuminated room. The stimuli were back-projected at 120 Hz with a resolution of 800 × 600 pixels onto a 1.25 × 1.0-m acrylic rear-projection screen (Techplex 15, Stewart Filmscreen Corporation, Torrance, CA, USA) tilted backward by 30°. Participants stood in front of the screen. An infrared camera (Optotrak 3020, Northern Digital) that was placed at about shoulder height to the left of the screen measured the position of a marker (an infrared light-emitting diode) attached to the nail of the index finger of the participant’s dominant hand at 500 Hz.

In order to synchronize the movement data (i.e., the marker position) with the stimulus presentation, the camera also recorded the position of a second marker attached to the side of the screen. This marker did not move but it stopped emitting infrared light so that its position was registered as “missing” when a flash was presented at the top left corner of the screen (where a light-sensor was placed to detect the flash). We used a simple four-point calibration to relate the position of the fingertip to the projected images, automatically correcting for the fact that the marker was attached to the nail rather than the tip of the finger.

### Stimulus and procedure

**Fig. 1:**
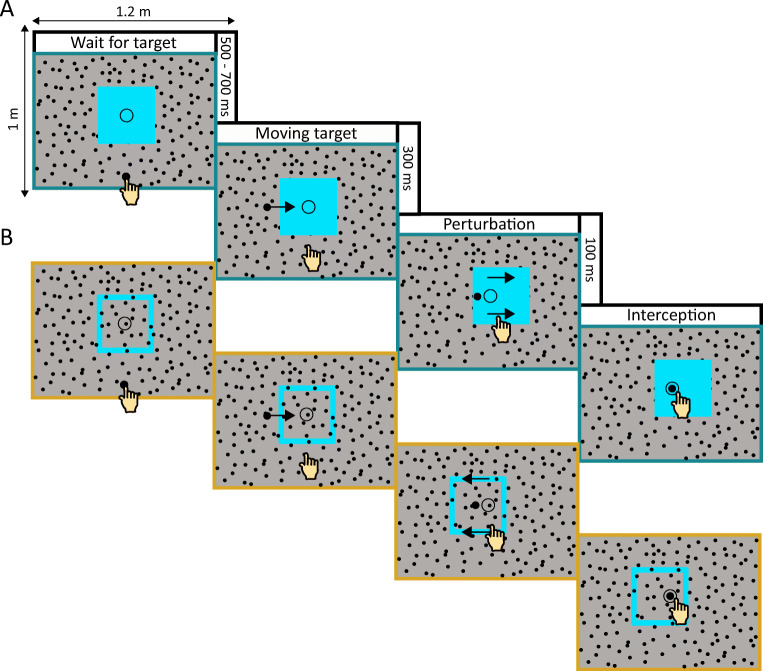
Timeline of the task. Participants had to tap the rightward moving target (black dot) when it reached the interception zone (indicated by the circle). The times given on the right denote the durations of each part of the task. (**A**) Display with a square tile that moves to the right. (**B**) Display with a square frame that moves to the left. The color of the display edge matches the color coding used in Figs. 2 and 3 to denote the different types of square

Participants stood in front of the large screen and were free to move as they wished. The display included four key components that were always visible (see Fig. 1B): a grey background, a layer of 600 randomly positioned 1-cm diameter black discs, a circle indicating the interception zone (6-cm diameter black ring) and a blue square (with sides of either 7, 37, or 67 cm). These components of the display remained static throughout the trial, apart from the blue square that moved at a fixed time on every trial. The interception zone was presented at the horizontal center of the screen 10 cm above the vertical center. The blue square was always initially centered on the interception zone. To give the impression that the interception zone was on the square, the square was a blue tile: the square was filled and occluded the layer of black dots. To give the impression that the interception zone was not on the square, the square was just a frame: only the outline was blue, and even the outline did not occlude the layer of black dots when they overlapped. The width of the frame was 5% of the width of the square. Importantly, in both cases the square’s motion only gives rise to changes in the image itself (i.e., motion signals) at the edges of the square, but for the tile the area at which people are supposed to hit the target is part of the moving surface, whereas for the frame it is not.

At the beginning of each trial, a black starting point (4-cm diameter disc) was presented 30 cm below the interception zone. To start a trial, participants placed the index finger of their dominant hand on the starting point. Between 500 and 700 ms after they did so, the starting point disappeared, and a black target (2-cm diameter disc) appeared 20 cm to the left of the center of the interception zone. This target moved rightward across the screen at 30 cm/s so that it reached the center of the interception zone 667 ms after it appeared. Participants were instructed to tap the target within the interception zone. From 300–400 ms after the target appeared the square moved either rightward or leftward at a constant speed of 20 cm/s (covering 2 cm during the 100 ms of motion). A short epoch of motion was used so that there was no motion at the time of interception, ensuring that the haptic feedback of no motion when participants hit the screen was consistent with the visual information (Cuijpers, Brenner, & Smeets, 2008; Schenk, 2012).

In order to provide participants with feedback on their hitting performance, we detected taps on-line. A tap was detected if the reduction in the distance to the screen between consecutive measurements decreased by more than 1 mm (i.e., a deceleration threshold of 50 m/s^2^) while the finger was less than 2 cm above the screen. If the position of the fingertip (as determined during calibration) was within the outline of the target, we considered the target to have been hit. If a target was hit it remained at the position at which it was hit for 500 ms. If the position of the fingertip was also within the interception zone, there was a sound indicating that the hit was successful. If a target was missed, it deflected away from the finger at 100 cm/s, also remaining visible for 500 ms unless it left the screen before that.

### Design

Two factors were manipulated in a within-subjects design: the type and size of the square. The square was either a frame or a tile (see Fig. 1). The lengths of the edges of the square were 7, 37, or 67 cm. The square moved on every trial. On half of the trials it moved leftward and on the other half of the trials it moved rightward. In total there were 25 leftward and 25 rightward trials for each of the six conditions (two types of square; three sizes) in each block. Participants completed two such blocks of 300 trials each, giving a total of 600 trials, in a single session that took approximately 45 min including a short break between the blocks. All conditions were randomly interleaved.

### Data analysis

To evaluate the time-course of the response to the motion of the squares, we first converted the measured lateral positions of the finger into lateral velocities by direct differentiation. This was done for every 2-ms interval for the first 300 ms after the square started to move (300–600 ms after the appearance of the moving target). For each participant, we then averaged the lateral velocity of the finger for every interval. We did so separately for trials in which the square moved leftward and ones in which it moved rightward. We subsequently determined the *response* by subtracting the average velocity for leftward motion from that for rightward motion. We did this for each condition of the experiment.

Trials with timing issues in the onset or offset of the square’s motion (< 1%) or with missing data (1%) were removed from the analysis. All other trials were included in the analysis, irrespective of performance. After determining the individual responses in each condition, the values were averaged across participants. We present the time-course of the responses to the motion signal as mean values with standard errors across participants. We quantified the *response magnitude* of the hand for each participant and condition by taking the mean response between 150 ms and 200 ms after the square started moving.

## Results

This experiment was not preregistered. All summary level data and the analysis code are available from the Open Science Framework repository at: https://osf.io/4vt59/?view_only=97c2e5cba7f545138695237d0b3d76aa. Participants hit the screen 662 ± 27 ms after the target appeared and hit the target within the interception zone on 70 ± 11% of the 600 trials (mean ± standard deviation across participants’ mean values).

**Fig. 2:**
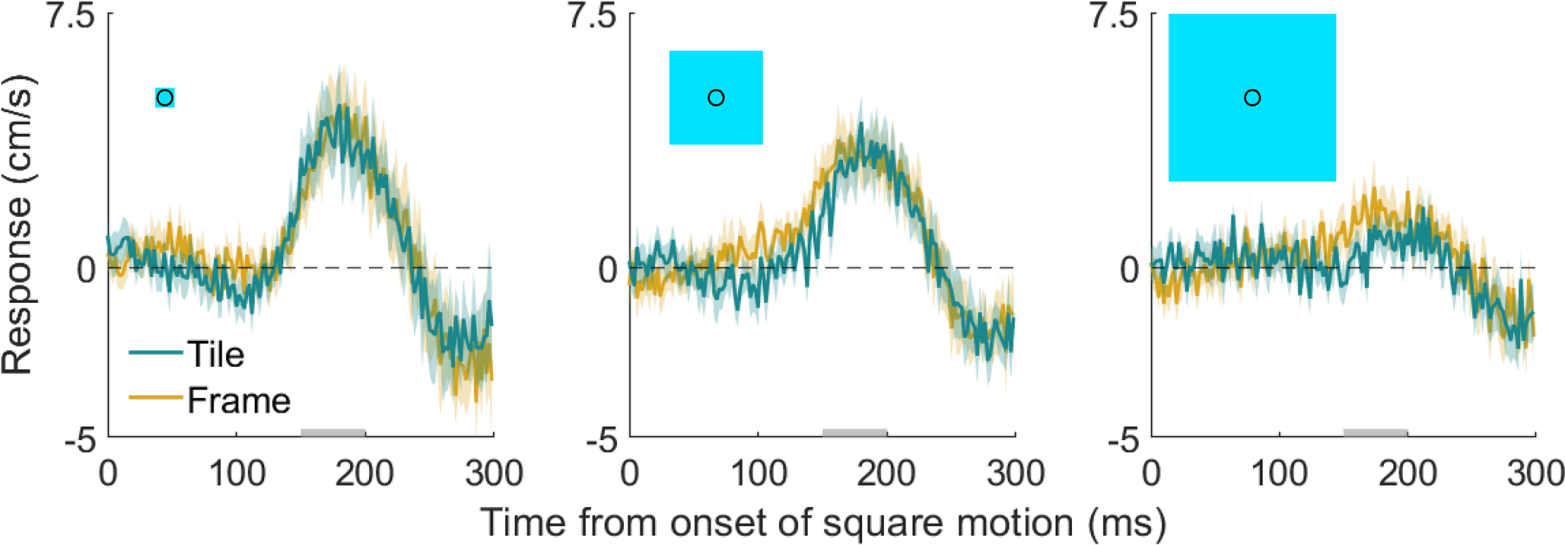
Time course of the hand’s response to the square’s motion. Each panel shows the data for a different square size (indicated by the sizes of the blue squares). Each curve shows the difference between the mean lateral hand velocity on leftward and rightward trials, averaged across participants. Shaded regions show the standard error of the mean across participants. A positive response is in the direction of the square’s motion. The grey bars at the bottom show the time-period with which we calculated each participant’s response magnitude

**Fig. 3:**
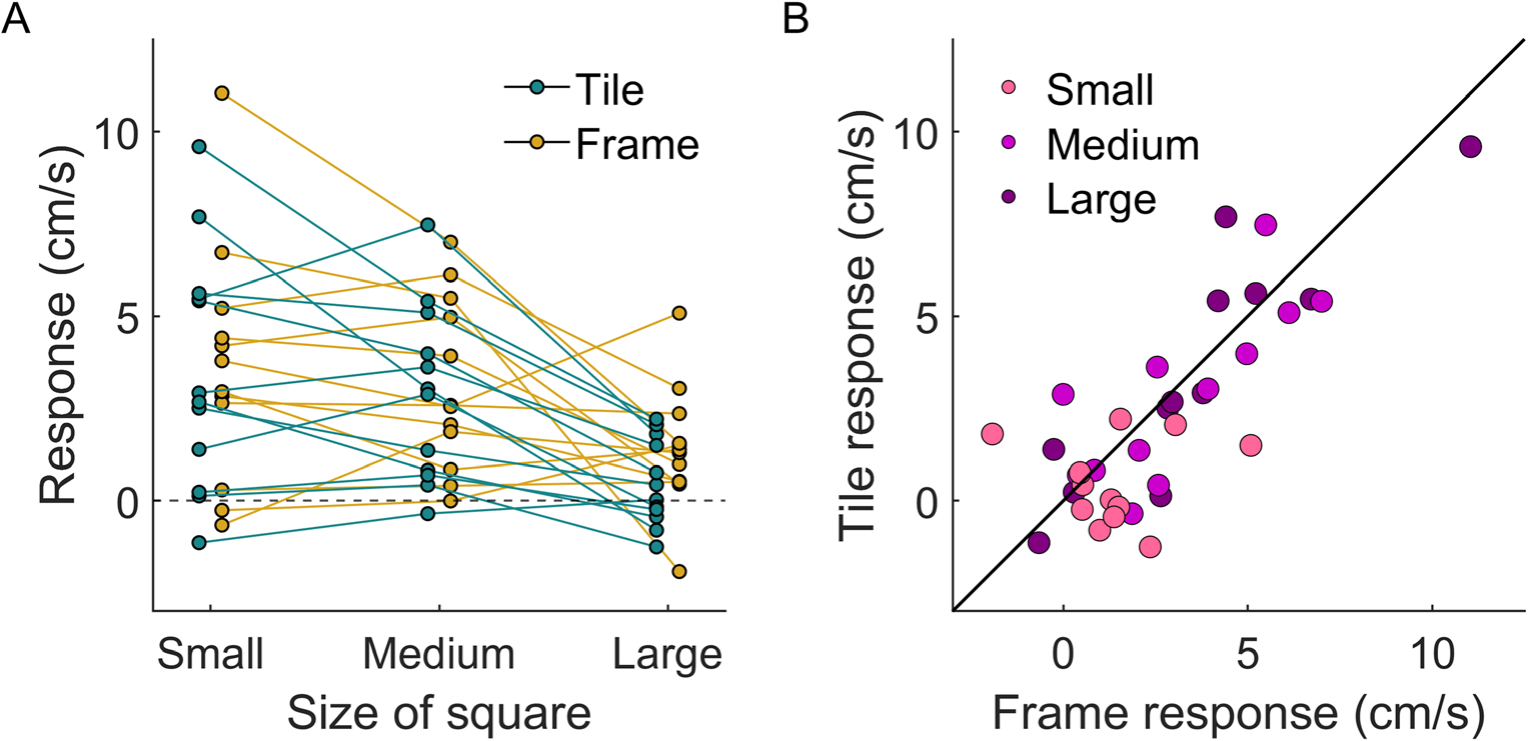
Each participant’s initial response (the average response between 150 ms and 200 ms from the onset of motion) to the two kinds of squares’ motion for each size of the square. (**A**) The data plotted as a function of the size of the square, with lines connecting individual participants’ data points for each kind of square. (**B**) The same data, now as the tile response plotted as a function of the frame response

The response of the hand (i.e., the difference in lateral hand velocity after leftward and rightward shifts) was similar for the two kinds of squares, evidenced by the almost perfectly overlapping golden and turquoise curves in Fig. 2. When the size of the square increases, the magnitude of the response decreases. This holds for most individual participants for both kinds of squares (Fig. 3A). The similarity of the response for the two types of squares was present for each combination of size and participant (Fig. 3B). The pattern of results is consistent with the response being driven by any motion near the endpoint of one’s action without considering whether the surface at which the movement ends is moving.

## Discussion

People continuously use visual information to adjust their movements in dynamic environments (Brenner & Smeets, 2018). For example, when reaching to a target item their movement is pulled in the direction of motion of the target (Brenner, Smeets, & De Lussanet, 1998; Brouwer, Middelburg, Smeets, & Brenner, 2003; de Lussanet, Smeets, & Brenner, 2001) but also by motion onset in the surrounding area of that item (e.g., Brenner & Smeets, 1997; Gomi et al., 2006; Saijo et al., 2005; Whitney et al., 2003; Zhang et al., 2018, 2019), presumably to stabilize the ongoing movement (Crowe et al., 2021). Since the visual system organizes its input into perceptually coherent objects and surfaces, it makes sense that this response to motion onsets might consider whether the surface at the anticipated movement endpoint is moving, and if not, how close the moving surface is to that endpoint. In this experiment, however, we found that the size of the response to motion onset was the same irrespective of whether the endpoint of one’s action was on a moving tile or a static background. The response was weaker when the motion signal was further away from the movement endpoint. These findings were consistent across the majority of participants. This suggests that the response does not consider whether the surface at which the movement ends is moving, but relies on the motion signal itself.

The similarity between participants’ responses to motion of the tile and of the frame clearly shows that the response did not consider the surface on which the endpoint of one’s action was located. The finding that the response is driven by the onset of any motion in the surrounding area, irrespective of what is moving, is consistent with finding a similar response when obstacles near the target move (Aivar, Brenner, & Smeets, 2008). The fact that in that case the response even occurred when a useful response would be in the opposite direction provides an additional argument against a more elaborate interpretation of the scene. The automaticity of the motion onset response is also consistent with research showing that the response is similar when the reaching task is performed in isolation and when it is performed alongside a second attentionally demanding counting task (De Dieuleveult, Brouwer, Siemonsma, Van Erp, & Brenner, 2018).

In line with other research (e.g., Brenner & Smeets, 2015; Crowe et al., 2021), we find evidence that the response is most sensitive to motion signals close to the endpoint of one’s action. This is in contrast to the conclusions drawn by Abekawa and Gomi (2010), who suggested that the response was not modulated by the location of the motion signal relative to the planned movement endpoint. They found no difference between the hand’s response to motion immediately surrounding the endpoint or separated from the endpoint by a grey mask occluding motion near the endpoint. These authors did not place the edge of the moving pattern further than 17 cm from the anticipated movement endpoint. Since we only found a small reduction in response magnitude when moving from the small- (3.5 cm) to the medium- (18.5 cm) sized squares in our experiment, these results are not too dissimilar from those reported by Abekawa and Gomi (2010). For our larger square size there was a clear decrease in the magnitude of the response. Even for our largest square we did see some response. It remains to be seen how distant the motion signal must be to abolish the response completely.

In this experiment, we find that the response to motion onset in the surrounding area of an ongoing movement does not take into consideration whether it is the surface at which the movement is planned to end that is moving. Instead, it is driven by a fast, automatic mechanism that detects and responds to any motion signal in the vicinity of the endpoint of one’s action. The response is modulated by the location of the motion signal relative to the endpoint of one’s action. It is smaller when the motion signal is further away, but whether the surface on which the movement endpoint is located could be interpreted as moving is irrelevant.

## Data Availability

The dataset generated and analyzed during the current study are available on the Open Science Framework Repository (https://osf.io/4vt59/?view_only=97c2e5cba7f545138695237d0b3d76aa).

## Code availability

The code used to analyze the data sets is available on the Open Science Framework Repository (https://osf.io/4vt59/?view_only=97c2e5cba7f545138695237d0b3d76aa).
